# Processing and Utilization of Functional Substances in Edible Plant Products and the Effects on Health Properties

**DOI:** 10.3390/foods12193648

**Published:** 2023-10-02

**Authors:** Zheling Zeng, Xianghui Yan

**Affiliations:** 1 State Key Laboratory of Food Science and Resources, Nanchang University, Nanchang 330047, China; 2 Jiangxi Province Key Laboratory of Edible and Medicinal Resources Exploitation, Nanchang University, Nanchang 330031, China; 3 School of Chemistry and Chemical Engineering, Nanchang University, Nanchang 330031, China; 4 School of Resources and Environment, Nanchang University, Nanchang 330031, China

## 1. Introduction

The world’s population is expected to reach 10 billion by 2050, which will pose a threat to the sustainable development of animal-derived foods. The excessive demand for animal-derived foods will directly contribute to freshwater depletion, climate change and biodiversity loss [1]. On the other hand, the production of animal-derived foods is more expensive in terms of energy and water resources than plant-derived foods. Moreover, from a health point of view, consuming a lot of meat in the diet is accompanied by high cholesterol and an increased saturated fatty acid intake, which may cause the occurrence of various chronic diseases such as obesity and cardiovascular disease. Hence, the demand for plant-based diets is expected to increase along with the growing population. This trend is also related to people’s concerns about health issues, as consuming plant-based diets is associated with health benefits in the body, such as antioxidant and anti-inflammatory activities.

Edible plants and plant products, both from conventional and non-conventional sources, are a noticeable part of the human diet. More importantly, edible plants usually contain a variety of macronutrients and micronutrients such as polyunsaturated fatty acids, proteins/peptides, polysaccharides, phenolic compounds, flavonoids, minerals, and vitamins. These nutrients are known to maintain the normal metabolism of the human body and have the effect of inhibiting the formation of chronic diseases. However, the range of commercially available edible plants and plant products is still very limited. Although thousands of edible plants have been discovered on Earth, only 150–200 have been utilized by humans so far [2]. Moreover, the structure–function relationships of many natural plant compounds remain unclear, which limits their application in the food and pharmaceutical industries.

## 2. Processing and Utilization of Edible Plants

The term “edible plants” refers to plants that can be consumed directly or used to produce food ingredients such as oils, proteins and polysaccharides. In the food industry, from raw plant materials to final food products, a series of unit operations are required, including material handling, cleaning, separation, crushing, liquid flow, mixing, heat transfer, concentration, drying, forming, packaging and process control. In particular, the interactions between food components (e.g., protein–phenolic interaction, carbohydrate–phenolic interaction and lipid–phenolic interaction) during food processing are not well understood [3,4]. On the other hand, plant components such as phenolic compounds are very sensitive to changes in the external environment (such as light and heat, etc.), and are easily degraded or oxidized to form quinones and lose their activity, which has serious impacts on their application in the industry. Moreover, most plant proteins cannot meet the requirements of food development and processing, due to their poor solubility, sensitivity to pH and temperature [5]. Hence, these unit operations need to be precisely controlled to obtain desirable color, flavor and texture in food. In the last few decades, researchers have been committed to exploring technologies that can improve the stability and functionalities of plant components. Different modification approaches, including physical, chemical and biotechnological processing, are being considered to improve the texture, nutritional value and shelf life of plant food products.

On the other hand, the processing of plant materials into food products may create numerous by-products or waste streams. Most plant by-products are characterized by a high level of low-cost nutrients, such as proteins, peptides, dietary fibers, polysaccharides and antioxidants (phenolics, flavonoids and vitamins). It is important to discuss the utilization of these by-products for food manufacture. Although much work has been carried out to explore the possibility of utilizing these by-products, only those recovery approaches that are environmentally friendly and sustainable will be considered.

## 3. Health Benefits of Edible Plants

One of the most important reasons to promote the consumption of edible plants is their health benefits. Edible plants are rich in bioactive compounds associated with a variety of biological activities, including regulating immune function, promoting growth, antimicrobial, antitumor and anti-inflammation activity, free radical scavenging, metabolic regulation, etc. Usually, in order to make more effective use of these biological actives, referred to as plant extracts, various technologies are required to separate and purify them from plants. Phenolic compounds are a class of important biologically active plant extracts, and more than 8000 structures of phenolic compounds have been found on Earth [6]. In recent years, in vitro gastrointestinal digestive models, cell models, and animal models have been widely used to evaluate the health benefits of plant extracts [7,8]. The potential value of plant extracts has been confirmed by many studies. However, given the structural diversity of bioactive compounds in plants, the mode of action of plant extracts is not fully understood. Therefore, the structure of bioactive compounds in plant extracts should be fully elucidated before evaluating their biological activities. In addition to HPLC-UV, HPLC-DAD, LC-ESI-MS, LC-ESI-QTOF-MS and LC-QQQ-MS, methodological improvement is still needed for the accurate qualitative and quantitative analysis of bioactive compounds in plant extracts. Moreover, it should be noted that plants of the same species in different geographical regions may differ in their biological activity due to changes in their chemical composition affected by environmental factors. Therefore, it is of significance to investigate the geographical pattern of valuable edible plants to provide a reference for plant breeding purposes.

## 4. Perspectives of Edible Plants

As mentioned above, edible plant resources that have been shown to be consumed by humans are very limited. Moreover, there is still much waste in the processing and utilization of edible plants. Therefore, it is hoped that awareness will be raised regarding agricultural biodiversity protection and land protection to avoid a reduction in edible plant species. At the same time, it is expected that more researchers will become involved in the high-value utilization of edible plants, the discovery of new sources of edible plants and the investigation of their relationship to health benefits.

Herein, this Special Issue focuses on the high-value utilization of edible plants, with respect to the processing and utilization of functional substances in edible plant products, as well as their effects on health properties.
